# Symptomatic Type IV Dual Left Anterior Descending Coronary Artery

**DOI:** 10.1177/2324709616683723

**Published:** 2016-12-01

**Authors:** Kyriacos Papadopoulos, Georgios M. Georgiou, Evagoras Nicolaides

**Affiliations:** 1 Medical Check-up Centre, Nicosia, Cyprus; 2 Apollonio Private Hospital, Nicosia, Cyprus; 3 University of Nicosia Medical School, Nicosia, Cyprus

**Keywords:** coronary artery anomaly, double left anterior descending artery, cardiac catheterization, exercise tolerance test, unstable angina

## Abstract

Dual left anterior descending coronary artery is a rare congenital anomaly with 4 subtypes. Double left anterior descending coronary artery originating from the left main stem and the right coronary artery (type IV dual left anterior descending artery) has been reported to occur in 0.01% to 0.7% of patients undergoing cardiac catheterization. We report a case of a 49-year-old woman who was found to have this anomaly during coronary angiography. The patient had been complaining of chest pain that mimics angina pectoris and exercise tolerance test was positive for myocardial ischemia.

## Introduction

The incidence of coronary artery anomalies ranges from 0.6% to 1.3% of patients undergoing coronary angiography.^1^

Among all of the coronary arteries, the left anterior descending (LAD) artery has the most constant course.^2,3^

Dual LAD artery is a rare anomaly. It consists of a short LAD artery that ends high in the anterior interventricular groove and a long LAD artery that most commonly originates as an early branch of the LAD artery proper (types 1-3) and rarely originates anomalously from the right coronary artery (RCA; type 4).

We report a case of a LAD artery originating from both right and left sinus of valsalva (type IV double LAD anomaly) that was discovered incidentally during cardiac catheterization.

## Case Report

A 49-year-old woman was admitted to the Cardiology Department of Nicosia General Hospital for scheduled cardiac catheterization because of angina on exertion and positive for myocardial ischemia exercise tolerance test (treadmill stress test).

Chest pain on exertion was the major complaint of the patient for the past 2 months. Arterial hypertension and diabetes mellitus were present as risk factors for coronary atherosclerosis.

Physical examination revealed a blood pressure of 126/78 mm Hg, with a regular pulse of 86 beats/minute. Cardiac auscultation revealed normal first and second heart sounds without murmurs or gallops. Lung sounds were clear.

An electrocardiogram showed normal sinus rhythm with no ST-T changes. Cardiac enzymes and routine blood investigations were normal.

Echocardiography showed normal left ventricular function with no wall motion abnormalities. Treadmill stress test revealed chest pain with ST depression in anterior leads at maximal exercise.

**Figure 1.**
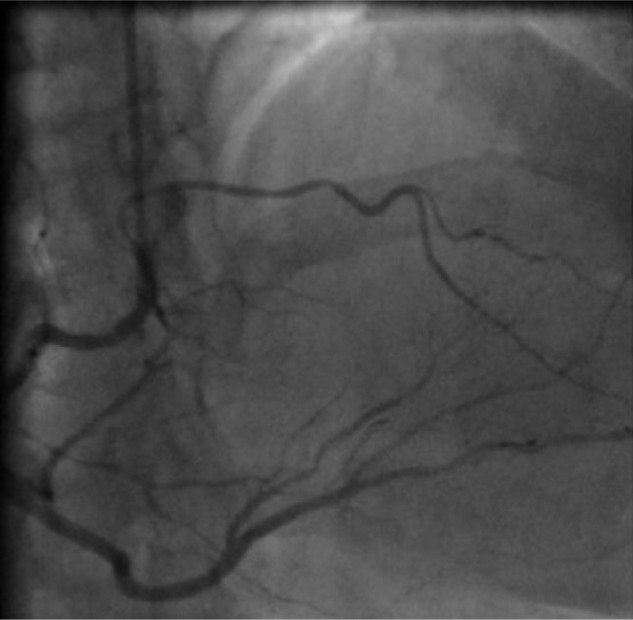
Selective coronary angiography of the right coronary artery (RCA) (RAO 30°) demonstrating that the left anterior descending (LAD) artery arose aberrantly from the right sinus of Valsalva and the proximal part of RCA (conus branch) supplying the area abandoned by the main LAD.

Coronary angiography was performed via the radial approach. Initially, the right coronary angiography revealed additional longer artery originating from the conus branch of the RCA (Figure 1).

**Figure 2.**
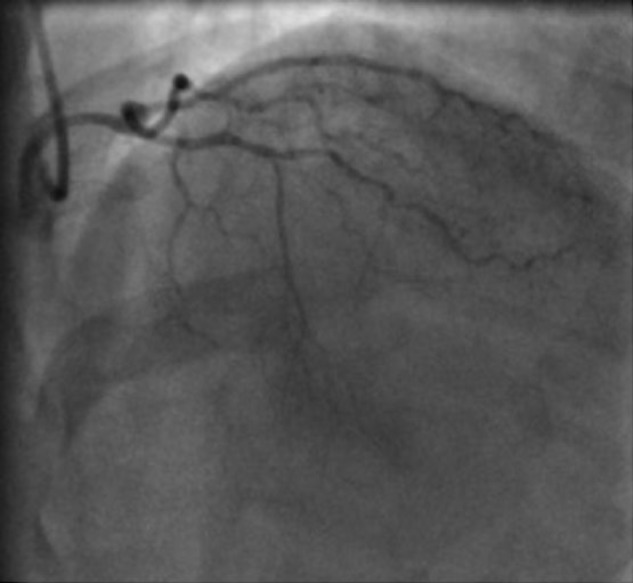
Left coronary angiography (RAO, Cranial) showing the left anterior descending artery ending at mid-segment without reaching the apex of the heart.

Contrast injection in the left coronary artery showed a small left circumflex without significant stenosis and the short LAD arising from the left coronary sinus, giving of 2 diagonal branches and terminating in the proximal interventricular groove (Figure 2).

This was consistent with the type IV variety of dual LAD, one of the rarest of coronary anomalies.

Systolic compression of the intraseptal segment that could account for our patient’s symptoms and abnormal exercise stress test was not evident.

Consequently, we arranged a computed tomography (CT) angiography in order to have a more accurate view of the course of the coronaries and their relationship with other structures.

## Discussion

The LAD normally arises from the left main coronary artery, courses in the anterior interventricular groove, and extends toward the cardiac apex. It gives rise to diagonal branches and septal perforators.

Anomalies involving the origin, course, and distribution of the LAD are rare, even though such variations are common with the RCA. Dual LAD (also known as dual anterior interventricular artery) has been known to occur with an incidence of 1% as described by Morettin^4^ as well as Spindola-Franco et al.^2^ Dual LAD can be associated with congenital heart disease such as tetralogy of Fallot and complete transposition of great arteries. This knowledge is very important at the time of the surgery.^5^

This anomaly is usually described by a short LAD usually terminating high in the anterior interventricular groove, whereas a long LAD after a proximal course outside the anterior interventricular groove returns to course in the groove in its distal course.

Dual LAD was classified by Moreno-Martinez et al^6^ as follows:

*Type I*: The short LAD courses in the anterior interventricular sulcus (AIVS) and gives rise to the major proximal septal perforators. The long LAD courses parallel to the short LAD in its proximal course on left ventricular side and reenters the anterior interventricular groove to supply the mid and distal parts of the left ventricle.
*Type II*: The long LAD descends over the right ventricle and then reenters the AIVS. The short LAD follows the same course as the one described in Type I.
*Type III*: The long LAD has a proximal intramyocardial course. The short LAD is the same as the ones described in Types I and II.
*Type IV*: The short LAD originates from the left main coronary artery, where the long LAD is a branch of the RCA taking an anomalous course and entering the anterior interventricular groove.

Other than this definition, Manchanda et al^7^ have described a novel variant of dual LAD, which they named type V dual LAD, where the short LAD originates directly from the left coronary sinus, and the long LAD originates directly from the right coronary sinus. Maroney and Klein^8^ also described a new case where a dual LAD is observed with the short LAD originating from the left main coronary artery and giving rise to the proximal septal perforator arteries and a single large diagonal artery. The long LAD originates from the RCA and traverses to the AIVS via a route underneath the right ventricular outflow tract. The long LAD gives off small septal perforator arteries in its proximal, mid, and distal segments. The authors propose that this is a novel variant dual LAD, which they called type V.^8^

Our case resembles type IV of dual LAD. This anomaly has been reported to occur in 0.01% to 0.7% of patients undergoing cardiac catheterization and is occasionally seen in the tetralogy of Fallot.^9^ In patients with tetralogy of Fallot, the origin of the LAD from the RCA is particularly important, since the LAD courses the potential site of surgical reconstruction

There are 3 variations in the initial course of LAD^9^:

1. Anterior to the right ventricular infundibulum (anterior type)
2. Between the aorta and the pulmonary trunk (intra-arterial type)
3. Within the ventricular septum beneath the right ventricular infudibulum (septal type)

According to this classification, our case is consistent with the septal type.

Multidetector row CT allows 3-dimensional comprehension of the coronary artery system and it is extremely useful to identify congenital coronary artery anomalies, regarding their origins, courses, and also relationships with other cardiac structures. CT is superior to coronary angiography in appreciating the precise course of the coronary arteries because of the omniplanar capability of CT.

In our patient, systolic compression of the intraseptal segment that could account for our patient’s symptoms and abnormal exercise stress test was not evident in coronary angiography. Consequently, we arranged a CT angiography in order to have a more accurate view of the course of the coronaries and their relationship with other structures.

Beside the clinical consequences of anomalous vessels, familiarity with the anatomic features of the coronary arteries is very important for planning surgical vascularization.^10^ The exact knowledge of the variants of dual LAD is crucial in order to avoid incorrect placement of an arteriotomy and revascularization of the wrong vessel.^5^ Additionally, in the case of the long LAD arising from the RCA, there is a risk of mistaking this type of coronary anomaly at routine coronary angiography for mid-LAD occlusion.

In conclusion, we presented a rare coronary anomaly known as double LAD type IV. It is important for cardiologists to be familiar with this variant of dual LAD anomaly when assessing coronary angiograms and making decisions about treatment.

## References

[1] Talanas G Delpini A Casu G Bilotta F Pes R Terrosu P A double left anterior descending coronary artery emerging from the right Valsalva sinus: a case report and a brief literature review. J Cardiovasc Med (Hagerstown). 2009;10(1):64-67. 19708227 10.2459/jcm.0b013e3283189350

[2] Spindola-Franco H Grose R Solomon N Dual left anterior descending coronary artery: angiographic description of important variants and surgical implications. Am Heart J. 1983;105:445-455. 6829406 10.1016/0002-8703(83)90363-0

[3] James TN Anatomy of the coronary arteries in health and disease. Circulation. 1965;32:1020-1033. 5846099 10.1161/01.cir.32.6.1020

[4] Morettin L Coronary arteriography: uncommon observations. Radiol Clin North Am. 1976;14:189-208. 823598

[5] Sajja LR Farooqi A Shaik MS Yarlagadda RB Baruah DK Pothineni RB Dual left anterior descendingcoronary artery: surgical revascularization in 4 patients. Tex Heart Inst J. 2000;27:292-296. 11093416 PMC101083

[6] Moreno-Martinez FL Vega LF Fleites HA Ibargollin R Gonzalez R Lopez OJ Dual left anterior descending coronary artery. Internet J Thorac Cardiovasc Surg. 2004;7(1). http://ispub.com/IJTCVS/7/1/11944. Accessed December 1, 2016.

[7] Manchanda A Qureshi A Brofferio A Go D Shirani J Novel variant of dual left anterior descending coronary artery. J Cardiovasc Comput Tomogr. 2010;4:139-141. 20430346 10.1016/j.jcct.2009.12.007

[8] Maroney J Klein LW Report of a new anomaly of the left anterior descending artery: type VI dual LAD. Catheter Cardiovasc Interv. 2012;80:626-629. 21953811 10.1002/ccd.23219

[9] Ebrahimi M Dargahy M Bajouri S Anomalous origin of left anterior descending coronary artery from right coronary artery associated with hypertrophic cardiomyopathy. Iran Heart J. 2008;9(2):59.

[10] Kheirkhah J Sadeghipour P Kouchaki A An anomalous origin of left anterior descending coronary artery from right coronary artery in a patient with acute coronary syndrome. J Tehran Univ Heart Center. 2011;6:217-219. PMC3467955 23074373

